# Destabilisation, aggregation, toxicity and cytosolic mislocalisation of nucleophosmin regions associated with acute myeloid leukemia

**DOI:** 10.18632/oncotarget.10991

**Published:** 2016-08-01

**Authors:** Pasqualina Liana Scognamiglio, Concetta Di Natale, Marilisa Leone, Roberta Cascella, Cristina Cecchi, Lisa Lirussi, Giulia Antoniali, Domenico Riccardi, Giancarlo Morelli, Gianluca Tell, Fabrizio Chiti, Daniela Marasco

**Affiliations:** ^1^ Department of Pharmacy, CIRPEB: Centro Interuniversitario di Ricerca sui Peptidi Bioattivi-University of Naples “Federico II”, DFM-Scarl, 80134, Naples, Italy; ^2^ Institute of Biostructures and Bioimaging - CNR, 80134, Naples, Italy; ^3^ Section of Biochemistry, Department of Experimental and Clinical Biomedical Sciences “Mario Serio”, University of Florence, 50134, Florence, Italy; ^4^ Laboratory of Molecular Biology and DNA repair, Department of Medical and Biological Sciences, University of Udine, 33100, Udine, Italy; ^5^ Permanent address: Center for Advanced Biomaterials for Health Care@CRIB Istituto Italiano di Tecnologia, 80125, Napoli, Italy; ^6^ Permanent address: Department of Clinical Molecular Biology, University of Oslo and Akershus University Hospital, Nordbyhagen, 1474, Norway

**Keywords:** helical peptides, aggregation phenomena, AML, CD spectroscopy

## Abstract

Nucleophosmin (NPM1) is a multifunctional protein that is implicated in the pathogenesis of several human malignancies. To gain insight into the role of isolated fragments of NPM1 in its biological activities, we dissected the C-terminal domain (CTD) into its helical fragments. Here we focus the attention on the third helix of the NPM1-CTD in its wild-type (H3 wt) and AML-mutated (H3 mutA and H3 mutE) sequences. Conformational studies, by means of CD and NMR spectroscopies, showed that the H3 wt peptide was partially endowed with an α-helical structure, but the AML-sequences exhibited a lower content of this conformation, particularly the H3 mutA peptide. Thioflavin T assays showed that the H3 mutE and the H3 mutA peptides displayed a significant aggregation propensity that was confirmed by CD and DLS assays. In addition, we found that the H3 mutE and H3 mutA peptides, unlike the H3 wt, were moderately and highly toxic, respectively, when exposed to human neuroblastoma cells. Cellular localization experiments confirmed that the mutated sequences hamper their nucleolar accumulation, and more importantly, that the helical conformation of the H3 region is crucial for such a localization.

## INTRODUCTION

Nucleophosmin 1 (NPM1, also known as B23.1, No38 and numatrin) is an abundant multifunctional protein belonging to the nucleoplasmin family of nuclear chaperones [1, 2]. This protein is present in high amounts in the granular region of nucleoli, taking part in rRNA maturation processes and it is essential for embryonic development. The N-terminal domain is the oligomerization domain mainly involved in its chaperone activity, it extends for approximately 100 residues and displays an eight-stranded β-barrel fold. The central portion of NPM1 is an IDR (Intrinsically Disordered Region) crucial for DNA/RNA recognition mechanism [3–5]. The C-terminal domain (CTD) forms a globular structure consisting of a three-helix bundle. NMR analysis showed that these three helices span over residues 243–259 (H1), 264–277 (H2), 280–294 (H3) [6].

The presence of several nuclear import/export signals in the NPM1 sequence indicates its propensity to act as a nuclear and/or nucleolar shuttling protein. Remarkably, it is involved in several biological processes such as ribosome biogenesis, tumor suppression and nucleolar stress response [7, 8]. While decreased NPM1 and NPM3 protein levels in the hippocampus at advanced stages of Alzheimer's disease have been described, resulting in nucleolar alterations and cell death [9], these proteins have been found over-expressed or mutated in tumors of different histological origin, including gastric, ovarian, bladder and prostate carcinomas as well as in various hematological malignancies [4, 5, 10]. Moreover, its incidence in the nucleoplasm or in other cellular compartments is documented in cells exposed to different types of stress [11, 12]. NPM1 has been identified as the most frequently mutated gene in acute myeloid leukemia (AML) patients, in particular specific mutations in the exon 12 of the NPM1 gene occur, accounting for approximately 30% of AML cases [2, 13–17]. Although these mutations occur exclusively in AML patients, their leukemogenic potential remains to be understood. It is known that they lead to the aberrant accumulation of the protein in the cytoplasm of the leukemic cells (hence the term NPM cytoplasmic positive NPMc+, for this AML subtype) [16]. The increased export of NPM1 into the cytoplasm affects multiple cellular pathways and could induce leukemia by either loss- or gain-of-function mechanisms: mislocation of NPM1 in the cytoplasm can cause the loss of its nuclear function and, simultaneously, its interactors can be delocalized into the cytoplasm, significantly impairing their functions. Additionally, NPM1 mutants acquire new properties such as the ability to interact and inhibit the cell death activity of caspase-6 and caspase-8 in the cytoplasm [18].

A so-called nucleolar localization signal (NoLS) is present in the three-helix bundle of NPM1. Importantly, although most of the identified NoLS are formed by sequence motifs enriched in consecutive basic residues, which are thought to be recognized by specific nucleolar hub proteins (including NPM1), the NoLS of NPM1 is unique and constituted by the two tryptophan residues in helix H3 [19]. AML-associated NPM1 mutations are small insertions of a different number of bases at the end of the gene, causing a frameshift and generating mutant NPM1 proteins with altered amino acid sequences. Depending on the mutation, the last 5–7 C-terminal residues ((WQ)WRKSL) are substituted by abnormal sequences of 9–11 amino acids [15]. These mutations lead to the loss of one or two Trp residues, as it is found in the type E and A mutated NPM1 proteins, respectively. It has been shown that loss of these residues, that are crucial elements of the hydrophobic core of the C-terminal domain, impairs the folding of the domain [6, 20] and hampers its ability for nucleic acids recognition [20–23]. Hence, mutated NPM1 proteins lose the NoLS [16] and, simultaneously, they acquire four additional amino acids at the C-terminus, which generate a leucine-rich nuclear export signal (NES) able to reinforce Crm1 (XpoI)-dependent nuclear export of NPM1 leukemic mutants [24]. These variations account for the aberrant and stable cytoplasmic localization of mutated NPM1 [1, 6, 25, 26]; indeed in AML patients with NPM1 mutations, the protein is largely found in the cytosol and only a limited portion is retained within nucleoli [1]. This feature characterizes this type of leukemia that has been included as a new provisional entity in the 2008 World Health Organization classification of myeloid neoplasms [14].

In order to gain insight into Structure-Activity Relationships (SAR) of isolated protein fragments, our previous studies were aimed at dissecting NPM1-CTD into three peptides corresponding to the three helices. In this way, we obtained information on their conformational behavior and their ability to interact with NPM1 biological partners [21]. The peptide corresponding to the H1 region does assume an α-helical structure, and, from NMR studies, helical content was found to be ~39% and stable upon increasing time and temperature; furthermore it appeared to be able to bind to G-quadruplex structures in a cooperative recognition along with its N-terminal IDRs [21]. On the other hand, the peptide encompassing the H2 region showed an unexpected remarkable tendency to form amyloid-like assemblies endowed with fibrillar morphology and β-sheet structure, under physiological conditions [27]. These aggregates appeared to be toxic in *in vitro* cell viability assays. Moreover, the extension of the H2 sequence beyond its N-terminus, including the linker loop connecting H2 with H1, delayed aggregation and its associated cytotoxicity [27].

Here, we combine cellular, biophysical and biochemical analyses to gain a deeper understanding of the molecular mechanisms involved in NPM1 subcellular localization, focusing the attention on the H3 region: this region was investigated as an isolated peptide in the wild-type sequence, as well as in the type A and type E AML-associated mutated sequences.

## RESULTS

Peptides corresponding to the H3 region of NPM1-CTD (H3 wt, H3 mutE and H3 mutA) were designed and, to avoid potential charge effects of the extremities, all peptides were acetylated and amidated at the N- and C-termini, respectively. All sequences are reported in Table 1. They were chemically synthesized with good yields by SPPS, using Fmoc methodologies and purified by RP-HPLC. Their identity and purity (averaged purity > 97%) were assessed by LC-MS (data not shown).

**Table 1:** Peptide sequences analyzed in this study, Leu and Val residues of AML associated NES are reported in bold

NAME	SEQUENCE	MW (amu)
H3 wt	^279^TDQEAIQDLWQWRKSL^294^ pI = 4.56	2060.2
H3 mutE	^279^TDQEAIQDLWQS**L**AQ**V**S**L**RK^298^ pI = 4.56	2373.5
H3 mutA	^279^TDQEAIQDLCLA**V**EE**V**S**L**RK^298^ pI = 4.18	2303.5
FITC-H3 wt	FITC-βAGRKKRRQRRRPPQGGTDQEAIQDLWQWRKSL	4292.2
FITC-H3 mutE	FITC-βAGRKKRRQRRRPPQGGTDQEAIQDLWQSLAQVSLRK	4604.3
FITC-H3 mutA	FITC-βAGRKKRRQRRRPPQGGTDQEAIQDLCLA**V**EE**V**S**L**RK	4535.7
FITC-H3 wt disordered	FITC-βAGRKKRRQRRRPPQGGGKGKPIQDLWQWRKSL	4215.2

### Conformational studies

**Figure 1:**
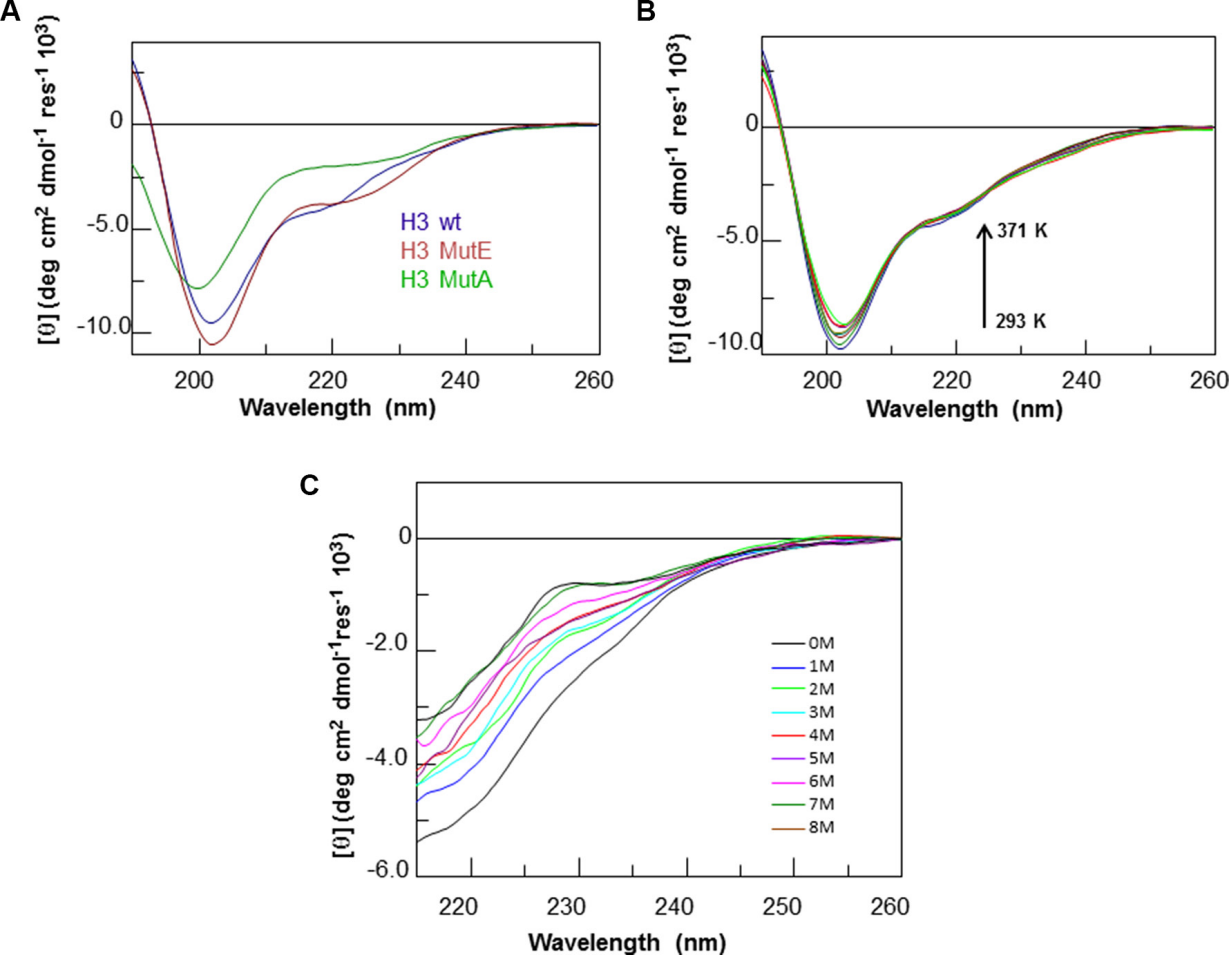
CD analysis of H3 derived peptides Overlay of CD spectra of (**A**) freshly prepared solutions of H3 derived peptides, (**B**) H3 wt at increasing temperature and (**C**) at increasing urea concentration.

To evaluate the structural preferences of the H3 variants in comparison with the wt H3 region within the native structure of the CTD, all peptides were characterized by far-UV circular dichroism (far-UV CD) and nuclear magnetic resonance (NMR) spectroscopies. Far-UV CD spectra were initially acquired in phosphate buffer at pH 7.2 (at 100 μM) for freshly prepared solutions (Figure 1A). The spectra acquired for H3 wt and H3 mutE indicate a mixture of unordered and α-helical conformations, as suggested by the presence of a strong negative band at ca. 202 nm, a weak negative band at 222 nm and positive values at ca. 190–193 nm. By contrast, the spectrum acquired for H3 mutA lacks a maximum at 190 nm, has a shift of the first minimum at ca. 200 nm and a reduction of the Cotton effect at 222 nm, indicating a mixed random coil and α-helical conformations in which random coil is highly predominant. These differences are in agreement with data reported on the entire wt CTD and mutated domains [6, 28].

As already reported for the H1 region of the NPM1-CTD [21], the α-helical conformation of the H3 wt peptide appeared stable within 4 h at 100 μM (data not shown) and upon increasing the temperature from 293 to 371 K, as shown by the overlay of the CD spectra collected over this range of temperature values (Figure 1B). This suggests that the H3 wt peptide retains a significant amount of the α-helical structure even at very high temperature, that is uncommon for short peptides. The analysis of the chemical stability of the H3 wt peptide was checked by using urea as a chaotropic agent (Figure 1C). In this case the ellipticity measured at 222 nm was significantly affected by the addition of even low amounts of the denaturant. A similar thermal and chemical stability was shown by the H3 mutE peptide (Supplementary Figure S1A and S1C), while the H3 mutA counterpart appeared to be more flexible, both following thermal and urea concentration variations (Supplementary Figure S1B and S1D).

2D-[^1^H, ^1^H] NMR spectroscopic analyses were performed for H3 wt, H3 mutE and H3 mutA peptides in 10 mM sodium phosphate, pH 7.2. Comparison of TOCSY and NOESY experiments allowed to get resonance assignments for most of the proton atoms (Supplementary Figure S2 and Supplementary Tables S1–S3). Generally, the propensity of a peptide to assume a certain type of secondary structure can be derived by analysis of Hα chemical shifts; this can be achieved by comparing the observed chemical shifts and random coil values [29, 30].

**Figure 2:**
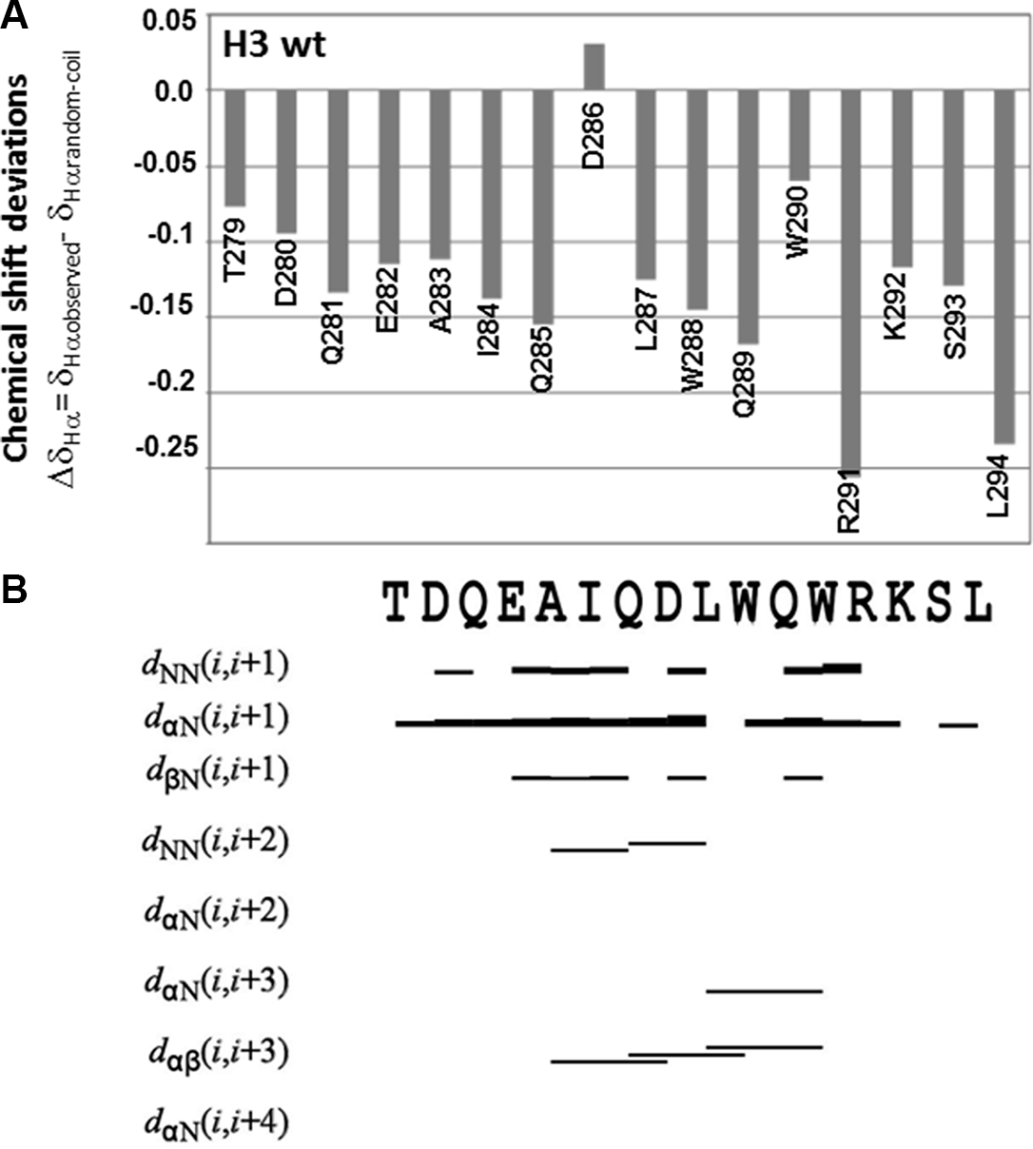
NMR secondary structure determination of H3 wt (**A**) Chemical shift deviations of Hα protons from random coil values (Δδ_Hα_ = δ_Hαobserved_ − δ_Hαrandom-coil_). (**B**) Summary of relevant sequential and medium-range NOEs. The NOE diagram was produced with the software CYANA version 2.1. Cross-peaks in the NOESY 300 spectrum were manually integrated. A NOE contact between the Hx and the Hy protons in the i and i + n residues is indicated as dHxHy_i, i+n_; the thickness of the bar is proportional to the NOE intensity. The primary structure is reported on the top of the graph. NMR data were estimated from a H3 wt sample (900 μM final concentration) in 10 mM sodium phosphate buffer pH = 7.2/D_2_O 95/5 v/v.

For the H3 wt peptide the chemical shift deviations from random coil values (CSD) appeared small, but negative for most residues, thus indicating a certain propensity towards α-helical conformations (Figure 2A). The percentage of α-helical content in the wild-type peptide was estimated from the CSD values (See Material and Methods for details) equal to 21%. The tendency of the H3 wt peptide to adopt α-helical conformation was confirmed by NOE analysis (Figure 2B), which revealed the presence of a few NOE contacts canonical for this type of secondary structure, such as Hα(Ala283)-Hβ(Asp286), Hα(Gln285)-Hββ′(Trp288), Hα(Leu287)-Hββ′(Trp290), Hα(Leu287)-HN(Trp290). A complete structure calculation was carried out with the software CYANA [31], which produced a structure with a pseudo α-helical conformation and tendency to adopt an unordered ensemble of conformations (Supplementary Figure S3). The majority of NMR conformers tend to adopt disordered bend elements in the segment encompassing residues Ala283-Arg291 that resemble helical turns. However, the number of relevant NOEs is still too small to produce a fully ordered and stable conformation. These structural calculations do not take into account possible aggregation phenomena that may occur at the high concentration used to perform the NMR experiments. However, it is worth noting that the absence of extensive line broadening in the NMR spectra does not point toward the presence of large H3 wt aggregates in solution.

**Figure 3:**
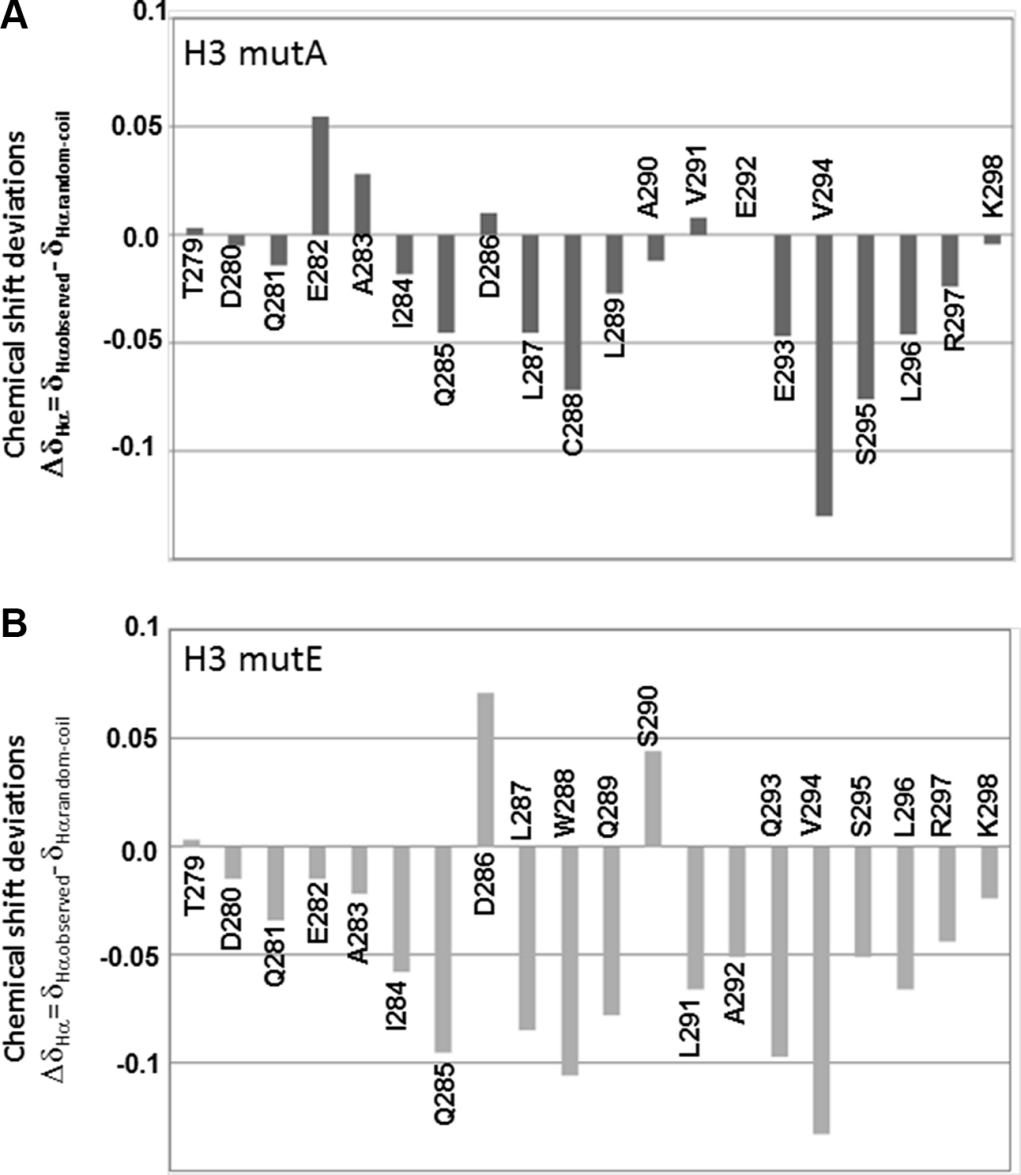
Chemical shift analysis of AML mutated sequences Chemical shift deviations of Hα protons from random coil values (Δδ_Hα_ = δ_Hαobserved_ − δ_Hαrandom-coil_) for (**A**) H3 mutA and (**B**) H3 mutE in 10 mM sodium phosphate buffer pH = 7.2/D_2_O 95/5 v/v at a final concentration of 900 μM.

We evaluated CSD values for H3 mutA and H3 mutE peptides as well (Figure 3A and 3B, respectively); negative deviations prevail for both peptides but their average values are rather small (lower than 0.1 ppm), particularly for the H3 mutA peptide, thus indicating a relevant amount of random coil content. The α-helical contents, evaluated for the H3 mutE and H3 mutA peptides from CSD data, were 16% and 10%, respectively. Analysis of NOESY spectra also confirmed prevalence of intra-residue and sequential contacts in mutated peptides (data not shown), which, however, were not numerous enough to determine a structure.

Overall, the far-UV CD and NMR analyses indicate that the H3 wt peptide is the most structured, adopting a mixture of α-helical and random coil structure. The two mutant peptides are less structured, particularly in the case of the H3 mutA peptide. The low chemical shift dispersions in the 2D [^1^H, ^1^H] spectra of the H3 mutE (Supplementary Figure S2B) and H3 mutA (Supplementary Figure S2C) peptides may point out to aggregation phenomena. Indeed, the NOESY spectrum of the latter is also characterized by line-broadening, typical of aggregated species (Supplementary Figure S2C).

### Aggregation studies

**Figure 4:**
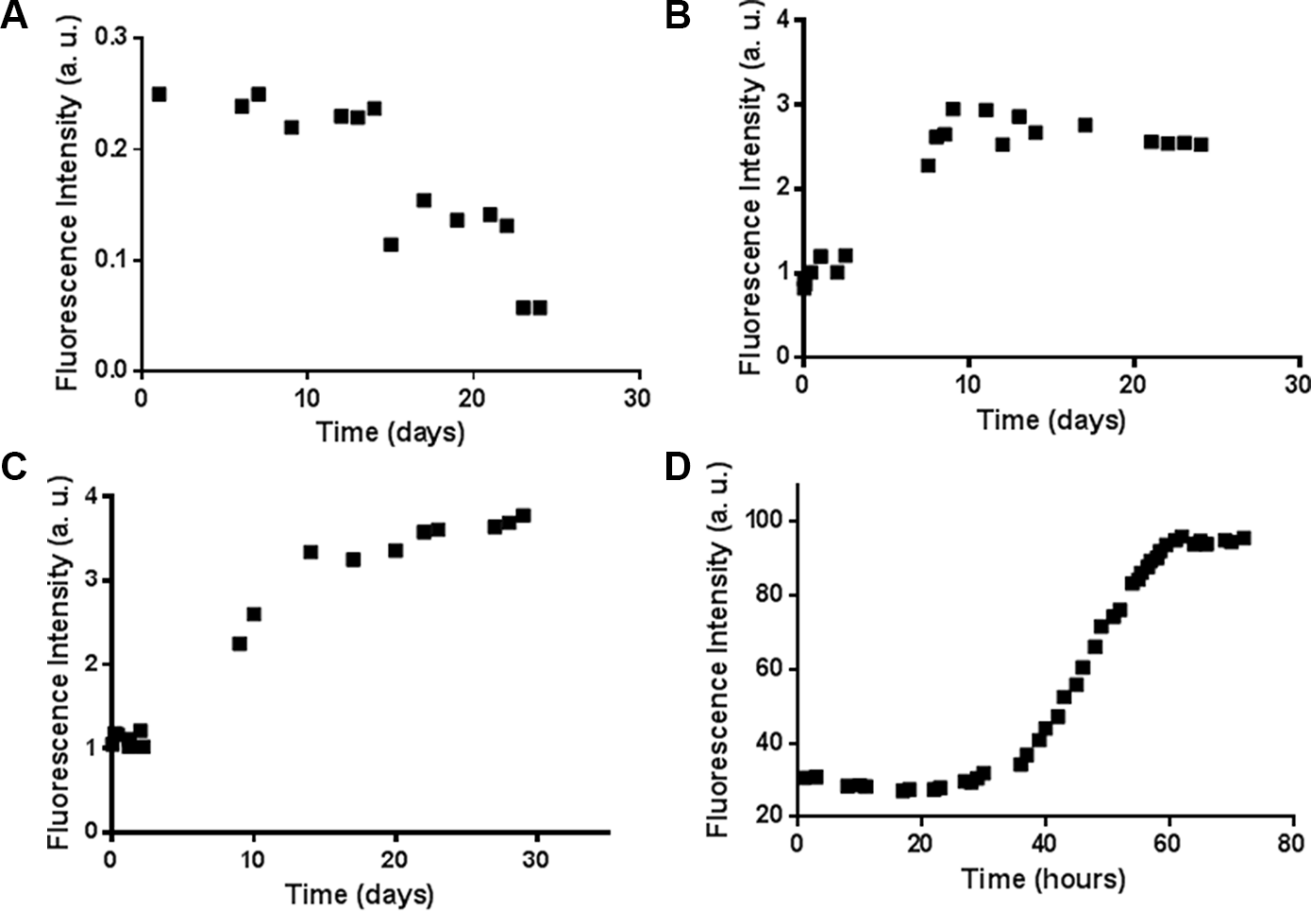
ThT assay for the H3 derived peptides Time course of ThT fluorescence emission intensity at 480 nm for the (**A**) H3 wt, (**B**) H3 mutE, and H3 mutA peptides at (**C**) 100 μM and (**D**) 2 mM.

Both the H3 mutA and H3 mutE peptides are expected to have an aggregation propensity higher than the H3 wt peptide, on the basis of the bioinformatic analysis carried out using the Zyggregator [32] and PASTA [33] algorithms (Supplementary Figure S4). To experimentally prove these predictions, the time course of Thioflavin T (ThT) fluorescence intensity in the presence of a freshly prepared solution of peptides was monitored at 100 μM, pH 7.2, under constant stirring. H3 mutE and H3 mutA peptides showed a clear time-dependent increase of ThT fluorescence intensity (Figure 4B and 4C), while the H3 wt peptide exhibited a weak, if any, higher value of ThT fluorescence at *t* = 0, which did not increase with time (Figure 4A). To further corroborate these results, a similar experiment was carried out at millimolar concentration for H3 mutA and the time dependence of ThT fluorescence is reported in Figure 4D. As expected for a nucleation-dependent growth mechanism, aggregation was faster reaching saturation within 60 hours.

**Figure 5:**
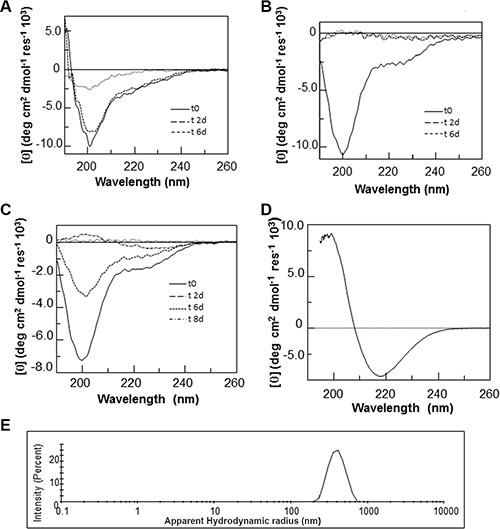
CD and DLS analyses of H3 derived peptides over time (**A**–**C**) Overlay of CD spectra of the (**A**) H3 wt, (**B**) H3 mutE, (**C**) H3 mutA peptides incubated at a concentration of 100 μM under stirring in 10 mM phosphate buffer, pH 7.2, and recorded at the indicated times. (**D**) CD spectrum of the H3 mutA peptide incubated at a concentration of 2 mM under stirring in 10 mM phosphate buffer, pH 7.2, and recorded after 15 days. (**E**) Size distribution of the H3 mutA sample at an equivalent monomer concentration of 2 mM recorded after 15 days.

With the aim of evaluating if the observed propensity to aggregate of H3 derived peptides is accompanied by a conformational transition, CD spectra of the H3-derived peptides were monitored at a concentration of 100 μM under stirring for 15 days. All H3-derived peptides showed a slow and progressive tendency to precipitate and/or aggregate over time, presenting a progressive decrease of the CD signal (Figure 5A–5C), as we already reported for the entire CTD mutA NPM1 [27]. The loss of signal for the H3 mutE and H3 mutA peptides was more rapid than for the H3 wt counterpart, indicating a more rapid aggregation process for the two mutated sequences. Moreover, CD spectra of all the H3-derived peptides were also monitored at a higher concentration of 2 mM under stirring and only the H3 mutA peptide exhibited a characteristic β-sheet far-UV CD spectrum with a broad minimum at 218 nm, after 15 days (Figure 5D).

The aggregation of the H3 peptides was further investigated by Dynamic Light Scattering (DLS) experiments over time at a peptide concentration of 2 mM. DLS is a quantitative, optical method for determining diffusion coefficients of particles undergoing Brownian motion in solution or suspension. The knowledge of diffusion coefficient of molecules allows the determination of size and flexibility of molecules. DLS is well suited for the detection of large aggregates such as amyloid since the intensity of the scattered light is proportional to the square of the particle mass [34]. As reported in Figure 5E, the H3 mutA peptide showed a correct DLS profile after 15 days with an apparent hydrodynamic radius centered at 400 nm. On the contrary, the H3 mutE and H3 wt peptides in the same conditions were not able to provide a correct correlation function, probably due to the presence of monomeric peptides or very small aggregates with hydrodynamic diameters less than the size limit of the instrument (data not shown).

### Cellular viability experiments

**Figure 6:**
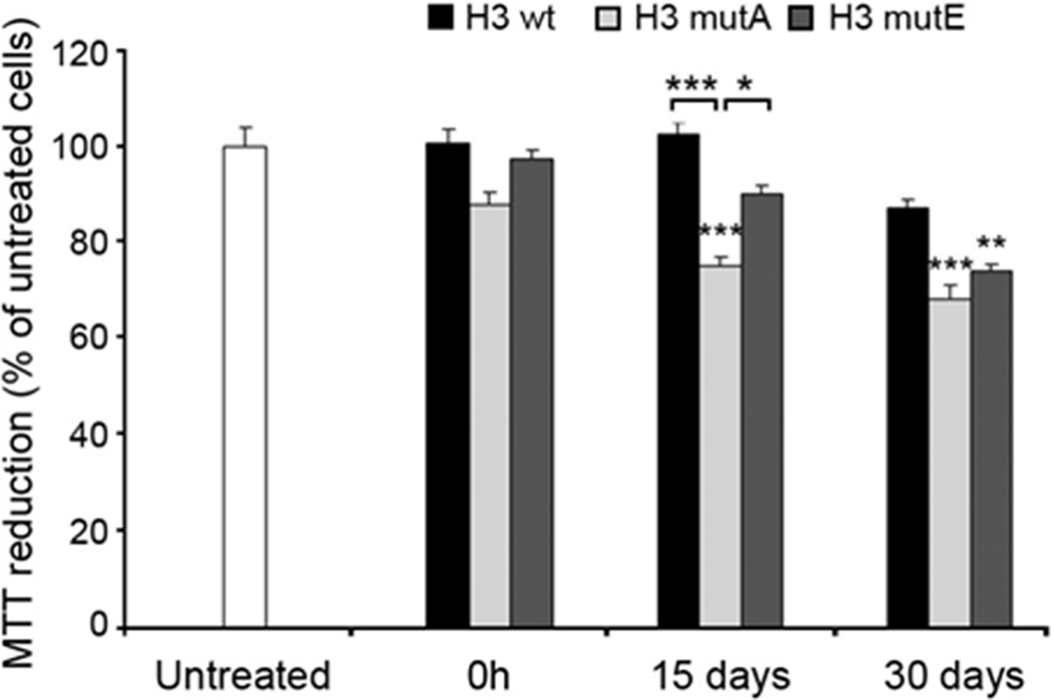
Cytotoxicity of the H3 derived peptides Peptides (1 mM monomer concentration) were incubated at 3 different times and then added to SH-SY5Y cells at a concentration of 100 μM. The values shown are means ± SEM of three independent experiments carried out in triplicate. The single, double and triple asterisks above each bar indicate significant differences (*p* ≤ 0.05, *p* ≤ 0.01, *p* ≤ 0.001, respectively) versus untreated cells, unless indicated otherwise.

The H3-derived peptides were also evaluated for their ability to impair cell viability. We first analyzed the effects of the aggregates formed from the H3 peptides on the mitochondrial status of human SH-SY5Y neuroblastoma cells, using the MTT reduction assay. The H3 wt, H3 mutA and H3 mutE peptides were incubated at a concentration of 1 mM in 50 mM phosphate buffer, pH 7.2, at 25°C, at 3 different times (0 h, 15 days and 30 days) and then added to the cells at a concentration of 100 μM. The three peptides added without pre-incubation (0 h) did not affect cell viability at this time-point (Figure 6). Following incubation for 15 days, the H3 mutA peptide appeared to be toxic; the H3 mutE peptide also appeared to be toxic but to a very weak and non-significant extent (Figure 6). By contrast, the H3 wt peptide was clearly nontoxic at this time. Overall, aggregates of the H3 mutA peptide showed a high toxicity at 15 and 30 days of incubation, aggregates of the H3 mutE peptide only after 30 days and the H3 wt peptide was found to be only weakly toxic at long times of incubation (Figure 6).

**Figure 7:**
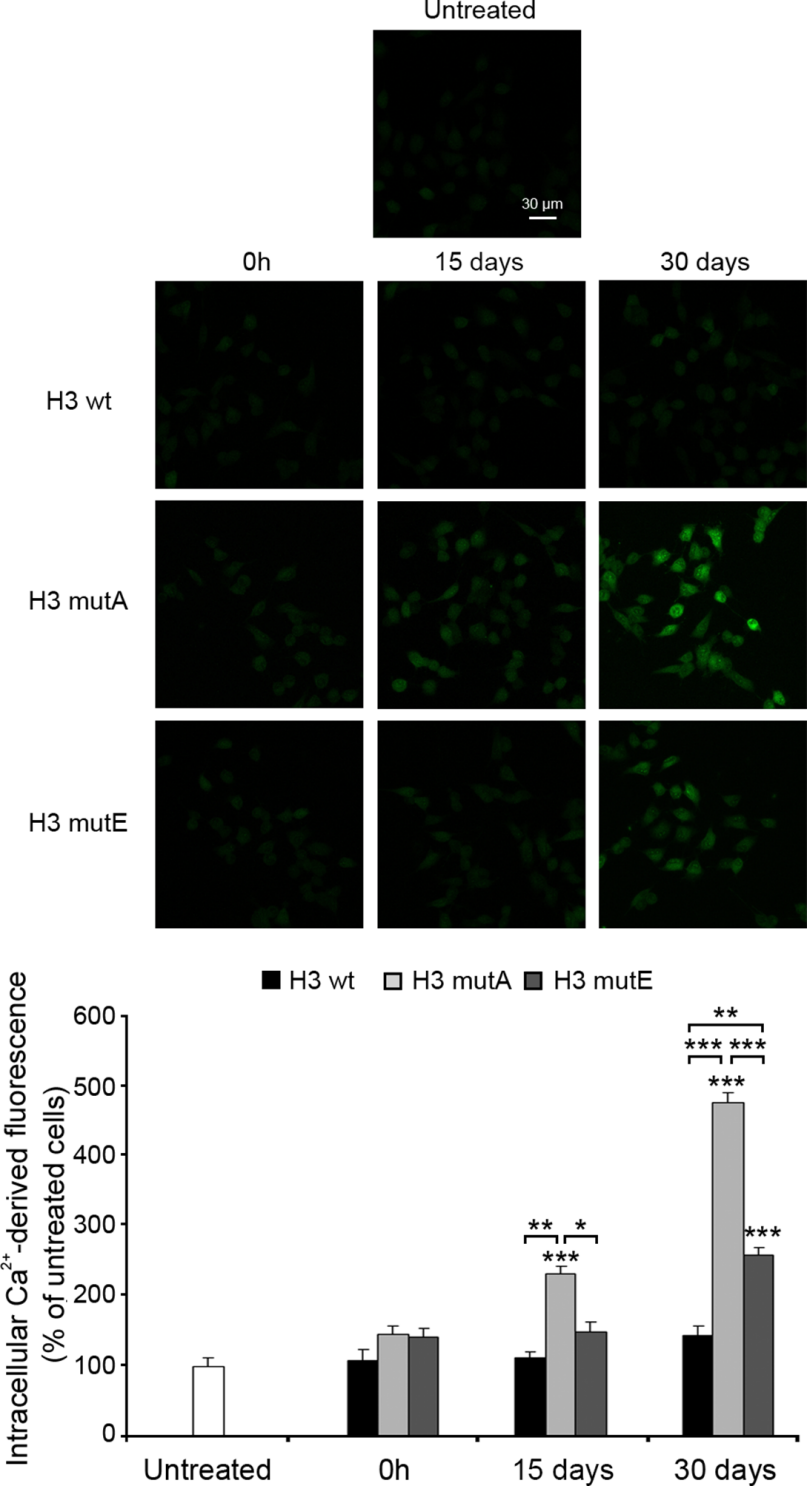
Rise in intracellular Ca^2+^ levels caused by the H3 derived peptides H3 derived peptides (1 mM monomer concentration) were incubated at 3 different times and then added to SH-SY5Y cells at a concentration of 100 μM for 60 min. The green fluorescence arises from the intracellular Fluo3 probe bound to Ca^2+^. The corresponding semi-quantitative values of the green fluorescence signals are shown below the images. The values shown are means ± SEM of three independent experiments carried out in triplicate. The single, double and triple asterisks indicate significant differences (*p* ≤ 0.05, *p* ≤ 0.01, *p* ≤ 0.001, respectively) versus untreated cells, unless indicated otherwise.

It is widely accepted that disruption of intracellular Ca^2+^ homeostasis is one of the earliest biochemical consequences of the interaction of prefibrillar aggregates with cell membranes [35–37]. We therefore investigated the effects of aggregated H3 peptides on the intracellular Ca^2+^ content in SH-SY5Y cells. The three peptides added without pre-incubation (0 h) did not cause any significant Ca^2+^ influx (Figure 7). Following incubation for 15 days, the H3 mutA peptide appeared to cause a significant Ca^2+^ influx; the H3 mutE peptide also had an effect but it was very weak and non-significant (Figure 7). By contrast, the H3 wt peptide did not affect Ca^2+^ homeostasis to any visible extent at this time. After 30 days of pre-incubation, both the H3 mutA and H3 mutE peptides appeared to be significantly toxic, whereas the H3 wt counterpart appeared only weakly toxic, with a Ca^2+^ influx that was non-significant relative to untreated cells (Figure 7). Overall, aggregates of the H3 mutA peptide were found to cause a large influx of extracellular Ca^2+^ ions into the cytosol at 15 days and, to an even higher extent, at 30 days of incubation; aggregates of the H3 mutE peptide caused an effect only after 30 days and the H3 wt peptide was found to be substantially harmless both after short and long times of incubation (Figure 7). In addition, the rise of Ca^2+^ influx caused by the aggregated peptides showed a trend and time dependency similar to that observed for the MTT reduction assay.

### Cellular localization experiments

NoLSs are short targeting sequences responsible for the localization of proteins to the nucleolus and are important regulatory elements controlling cellular traffic. In NPM1, the NoLS motif is so unusual that is not recognized as such by specific algorithms such as NOD (Nucleolar localization sequence detector) (http://www.compbio.dundee.ac.uk/nod) whose analysis is reported in Supplementary Figure S5. In order to evaluate the H3-derived peptides' subcellular distribution, cell transfection experiments and confocal analysis imaging were performed.

We conducted cellular experiments using the three H3 derived peptides conjugated to the CPP (Cell Penetrating Peptide) corresponding to the fragment 48–60 of the HIV Tat protein carrying Fluoresceine isothiocyanate (FITC) as a fluorophore (Table 1). It has been reported that this sequence, once expressed alone in HeLa cells, is diffusely scattered in the cytosol and, to lesser extent, within the nucleus, depending on time upon transfection [38]. Furthermore, we hypothesized on the importance of the wild-type spacing of tryptophan residues for the nucleolar localization and of their involvement in a well-defined secondary structure. Since the NMR analysis pointed out to the involvement of both tryptophan residues in a helical turn, we designed a variant of the H3 wt peptide (named H3 wt disordered) bearing the wild type spacing of Trp^288^ and Trp^290^ at the C-terminal extremity but with different residues in the N-terminal part with a very low predicted α-helical content, according to Agadir [39] (Table 1).

**Figure 8:**
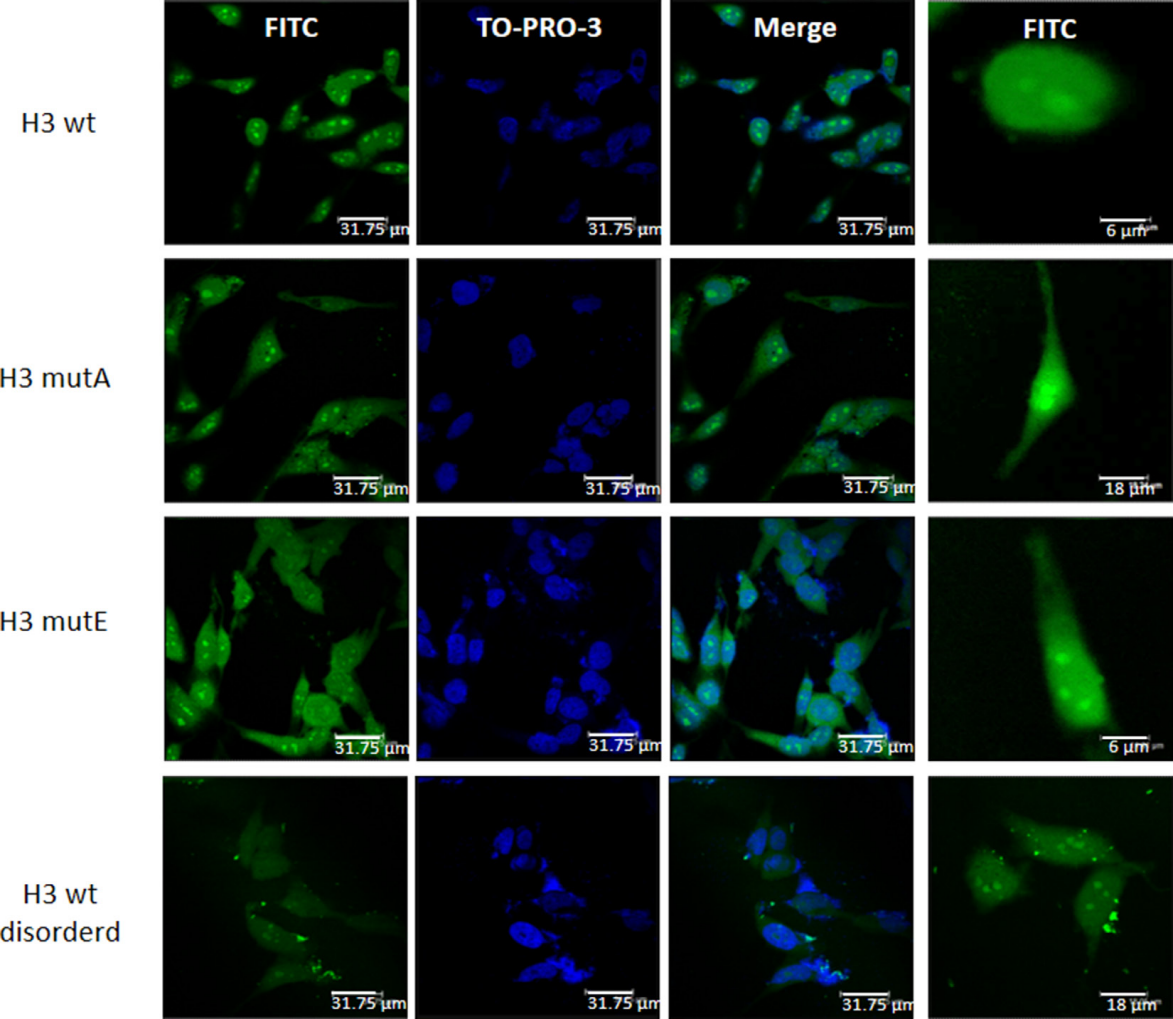
Subcellular localization the H3 derived peptides Representative fluorescence microscopy images showing the differential subcellular localization of different H3 derived peptides conjugated with a CPP and FITC (H3 wt, H3 mutA, H3 mutE and H3 wt disordered) in HeLa cells. Nuclei are counterstained with TO-PRO-3; bars, 31.75 μm. The image is representative of all transfected cells, of three independent biological replicas, in the field. Left panels show an higher magnification of single cells for each condition.

HeLa cells were transfected with FITC-containing H3 derived-peptides and nuclear staining was detected with TO-PRO-3 (Figure 8). The H3 wt peptide localized mainly in the nuclei with a clear accumulation within the nucleoli. For the H3 wt disordered peptide a pancellular distribution could be appreciated with no apparent accumulation in any compartment, supporting that the specificity of the nuclear localization depends on a well-defined structural conformation. Both H3 mutated peptide sequences resulted distributed between the nuclear and the cytoplasmic compartments, with a significant accumulation within this latter compartment in all the transfected cells.

## DISCUSSION

AML encompasses a heterogeneous group of diseases that has traditionally relied on morphology, cytochemistry and cytogenetic analysis for classification into various subtypes and in turn into different prognostic categories. The AML genome contains more than 20 driver recurrent mutations. The classification of AML into prognostic subgroups depending on the specific gene mutations, the so-called “AML with gene mutations”, includes mutations in Fms-like tyrosine kinase 3 (FLT3), nucleophosmin 1 (NPM1) and chloramphenicol acetyltransferase-box enhancer-binding protein alpha (CEBPA) [40]. All resulting mutant NPM1 translation products change their nuclear localization signal and a shift in the balance of nuclear export occurs, leading to the accumulation of NPM1 in the cytoplasm [15, 41–43].

AML cells carrying NPM1 mutations always retain a certain amount of wild-type NPM1 in the nucleolus [16]. This is probably necessary for the survival of leukaemic cells [44], since NPM1 mutations in AML are always heterozygous and knock-out mice with complete deletion of the NPM1 gene die during early embryogenesis [10]. Because NPM1 acts in the nucleolus as a hub building protein [45], the nucleolus of NPM1-mutated AML cells may be more vulnerable with respect to cells expressing only wild-type NPM1. Indeed AML cells are partially depleted of NPM1 as a consequence of both NPM1 haploinsufficiency and cytoplasmic delocalization of wild type NPM1 protein as complexes with the mutants. Thus, mutation-induced changes of the levels or the oligomerization status of NPM1 may result in defective assembly of the nucleolus in *NPM1*-mutated AML cells, in addition to affecting its functionality and the interaction with protein partners. The mechanism through which cytoplasmic nucleophosmin contributes to leukaemogenesis remains mainly unknown, but recently it has been reported that NPM1 mutations are involved in leukemia cell viability and invasion and that Matrix metalloproteinases (MMPs) regulated by the K-Ras/ERK MAPK signaling pathway play a role in this process [46].

Furthermore, NPM1 mutations are stable since they are detected during the whole course of the disease including relapses and thus represent a suitable target for immunotherapy. Indeed, NPM1 mutated sequences have been recently studied for their immunogenic properties associated with cytoplasmatic localization and for the development of immunotherapy for the treatment of AML patients [47–49].

Our recent study outlined the ability of the second helix of the CTD of NPM1, H2, to aggregate in an amyloid-like manner [27]. We hypothesized that the structural destabilization of the CTD in NPM1-AML mutants may favor the aggregation through the exposure of the amyloidogenic H2 region. Thus, to gain insight into the molecular basis of NPM1 transport in physiological and pathological conditions, we have carried out a biophysical and biochemical study of the third helix of the CTD, H3, in wild type as well as AML-mutated versions. A combined CD and NMR analysis shows that the region of the sequence corresponding to H3 wt partially adopts an α-helical conformation, despite its intrinsic flexibility. The NMR analysis shows that the whole H3 wt peptide (residues 279–294) exhibits 21% α-helical structure and that the segment from Ala^283^ to Arg^291^ exhibits a tendency to adopt an α-helical structure, involving Trp^288^ and Trp^290^. CD data indicate that this helical segment is rather stable upon temperature increase, that is unusual for a short peptide directly derived from a protein region but was also observed for the region corresponding to H1 of the CTD [21]. CD and NMR data indicate that AML-associated mutations of H3 (mutE and mutA sequences) confer the H3 region further flexibility that hampers α-helical formation and favors aggregation.

Algorithms that predict the aggregation propensity profile of a given sequence suggest the presence of another region in NPM1-CTD able to aggregate, in addition to H2: the H3 sequences of the leukemic type A and type E mutations of NPM1. Our *in vitro* aggregation results on H3 derived peptides and their cytotoxicity confirm that the mutated forms of H3 have a higher propensity to aggregate than the wt sequence and generate toxic aggregates. Hence, the two mutations of type A and type E can induce aggregation of the whole CTD by increasing the intrinsic aggregation propensity of the H3 region, as well as by destabilizing the whole C-terminal domain exposing the aggregation potential of the H2 region. Thus, the aggregation of the NPM1 mutant A and E could be driven from these two distinct regions, H2 and H3, that would explain the cellular accumulation phenomena with a loss of protein function.

Overall, our results show that H3 wt does not evolve towards amyloid structures for both its ordered helical structure and its low propensity to aggregate. By contrast, both the mutations present in H3 mutE and mutA sequences cause a partial and a complete loss of conformational order, respectively, do not alter the pI of the peptides and presumably the electrostatic interactions among monomers, and introduce new hydrophobic residues (such as Leu and Val) that, in addition to representing new NES motifs interacting with exportin CRM1, cause major insolubility that prompts aggregation in disordered states. Furthermore, in mutated sequences new H-bonding residues appear (Ser in mutE, Glu in mutA) that may possibly favour intermolecular interactions and aggregration. This effect is more apparent in the case of Glutamic residues of mutA with respect to Serine residues in mutE.

Moreover, the loss of the unusual aromatic NoLS in the H3 region following mutation [50] and the generation of a new nucleus export signal (NES) [24], explain why NPM1 is translocated from the nucleolus to the cytoplasm. Both tryptophan residues appear to be important for nucleolar localization, with Trp^290^ being more critical since it is mutated in all the so far identified NPM mutants. Furthermore, maximal inhibition of nucleolar binding and nucleoplasmic delocalization of mutants are observed when both tryptophans are mutated [50]. However the mutant sequences did not show a fully cytoplasmatic localization. The acquired NES in mutA and mutE peptides even if able to recognize CRM1 alone, as recently reported [24], need to cooperate with other NES motifs within the full-length sequence of NPM1 (residues 42–61 and 94–102) to fully localize in the cytoplasm the entire NMP1-AML associated proteins. Our cellular localization experiments also show that the α-helical conformation of the H3 region is important in this regard. Hence, the importance of the tryptophan residues also within short peptide sequences is once again proved and the secondary structure in which they are involved is crucial for specific nuclear localization. These results highlight our hypothesis that the cytosolic accumulation of mutated protein can be due to (i) aggregation events of the C-terminal domain (ii) and loss and gain of NoLS and NES signals, respectively.

In conclusion, our results indicate that the two AML-associated mutations destabilize the α-helical structure of the segment encompassing the H3 α-helix in the native NPM1-CTD and predispose it to formation of toxic aggregates. In addition, such H3 destabilization may contribute to that of the entire NPM1-CTD with consequent exposure of the H2 region, which is by far the most amyloidogenic region of the whole NPM1-CTD. The results also corroborate the hypothesis that the two mutations cause the mislocalisation of the NPM1 protein from the nucleolus to the cytoplasm and that such as a cytosolic accumulation is accompanied by aggregation events of the CTD and hetero-oligomerization of the N-terminal domain.

## MATERIALS AND METHODS

### Peptide synthesis

Reagents for peptide synthesis (Fmoc-protected amino acids and resins, activation and deprotection reagents) were from Novabiochem (Laufelfingen, Switzerland) and InBios (Napoli, Italy). Solvents for peptide synthesis and HPLC analyses were from Romil (Dublin, Ireland); reversed phase columns for peptide analysis and the LC-MS system were from Thermo Fisher (Milan, Italy). Solid phase peptide syntheses were performed on a fully automated multichannel peptide synthesizer Syro I (Multisynthech, Germany). Preparative RP-HPLC were carried out on a Shimadzu LC-8A, equipped with a SPD-M10 AV detector and with a Phenomenex C18 Jupiter column (50 × 22 mm ID; 10 μm). LC-MS analyses were carried out on a LCQ DECA XP Ion Trap mass spectrometer equipped with a OPTON ESI source, operating at 4.2 kV needle voltage and 320°C with a complete Surveyor HPLC system, comprised of MS pump, an autosampler and a photo diode array (PDA). Narrow bore 50 × 2 mm C18 BioBasic LC-MS columns were used for these analyses.

The peptide sequences reported in Table 1 were synthesized employing the solid phase method on a 50 μmol scale following standard Fmoc strategies [51]. Rink-amide resin (substitution 0.5 mmol/g) was used as solid support. Activation of amino acids was achieved using HBTU/HOBt/DIEA (1:1:2), whereas Fmoc deprotection was carried out using a 40% (v/v) piperidine solution in DMF. All couplings were performed for 15 min and deprotections for 10 min. At the end of peptide chain assembly, all peptides were acetylated at their N-termini. Peptides were removed from the resin by treatment with a TFA:TIS:H2O (95:2.5:2.5, v/v/v) mixture for 90 min at room temperature; then crude peptides were precipitated in cold ether, dissolved in a water/acetonitrile (1:1, v/v) mixture and lyophilized.

Products were purified by RP-HPLC applying a linear gradient of 0.1% TFA CH_3_CN in 0.1% TFA water from 5% to 65% over 12 min using a semi-preparative 2.2 × 5 cm C18 column at a flow rate of 20 mL/min. Peptides purity and identity were confirmed by LC-MS. To perform cellular assays, the fragment 48–60 of the HIV Tat protein as CPP (Cell Penetrating Peptide) was conjugated to peptides in a stepwise manner and purified (Table 1). These conjugated peptides were labelled with Fluorescein-βAla at their N-termini. Purified peptides were lyophilized and stored at −20°C until use.

### CD spectroscopy

CD spectra were recorded on a Jasco J-810 spectropolarimeter (JASCO, Tokyo, Japan) using a 0.1 cm path-length quartz cuvette. CD spectra were registered at 25°C in the far UV region from 190 to 260 nm. Each spectrum was obtained averaging three scans, subtracting contributions from corresponding blanks and converting the signal to mean residue ellipticity in units of deg cm^2^ dmol^−1^ res^−1^. Other experimental settings were: 20 nm/min scan speed, 2.0 nm band width, 0.2 nm resolution, 50 mdeg sensitivity, and 4 sec response. The concentration of peptides was kept at 100 μM in 10 mM phosphate buffer, pH 7.2. Millimolar samples were centrifuged.

Thermal variation profiles were obtained by measuring the temperature dependence of the ellipticity at 222 nm in the 293–371 K range with a resolution of 0.5°C and 1.0 nm bandwidth. The heating rate was 1 K/min and the response at 16 s with a Peltier temperature controller. A buffer solution containing 10 mM of sodium phosphate was used in chemical denaturation experiments. Urea was purchased from Sigma and further purified by recrystallization from ethanol/water (1:1) mixtures. Stock solutions of urea were mixed with peptide solutions to give a constant final value of the peptide concentration (100 μM). The final concentration of denaturant was in the range 0.0–8.0 M. Each sample was incubated overnight. Longer incubation times led to identical spectroscopic signals.

### NMR spectroscopy

NMR analysis was performed at 298 K on a Varian Unity Inova 600 MHz spectrometer provided with a cold-probe. NMR samples consisted of peptides (H3wt, H3mutA and H3mutE) dissolved in 600 μL of a mixture 10 mM sodium phosphate buffer pH = 7.2/D_2_O (99.8% d, Armar Scientific, Switzerland) 95/5 v/v at a final concentration of 900 μM.

The process of proton resonance assignments was carried out with the canonical Wüthrich procedure [52] based on combined analysis of 2D [^1^H, ^1^H] TOCSY (Total Correlation Spectroscopy) (70 ms mixing time) [53] and NOESY (Nuclear Overhauser Enhancement Spectroscopy) (200 and 300 ms mixing times) [54] experiments. TSP (Trimethylsilyl-3-propionic acid sodium salt-d4,99% d, Armar Scientific, Switzerland) was used as internal standard for chemical shifts referencing (See Supplementary Tables S1–S3 for H3 wt, H3 mutA and H3 mutE, respectively).

1D spectra were acquired with a relaxation delay of 1s and 32–64 scans. 2D [^1^H, ^1^H] experiments were usually recorded with 32–64 scans, 128–256 FIDs in t_1_, 1024 or 2048 data points in t_2_. Water suppression was achieved by *Excitation Sculpting* [55]. The software VNMRJ (Varian/Agilent Technologies) was implemented for spectra processing; the program NEASY [56] (included in the CARA (Computer Aided Resonance Assignment) software package) (http://www.nmr.ch/)) was used for spectra analysis.

Chemical shift deviations from random coil values for Hα protons (CSD) were calculated with the protocol suggested by Kjaergaard and collaborators by keeping into account the influence of neighbouring amino acids (http://www1.bio.ku.dk/english/research/pv/sbin_lab/staff/MAK/randomcoil/script/) [29, 30, 57].

Helical populations were estimated from CSD data with the equation: [Δδ_Hαaverage_/(−0.39)] × 100 where [(CSD = Δδ_Hα_= δ_Hαobserved_− δ_Hαrandom-coil_)] was averaged over all residues in a helical conformation [58]. Peptide structure calculations were conducted with the software CYANA [31] (version 2.1). Distance constraints for structure calculations were generated from a NOESY 300 ms experiment. Angular constraints were produced with the GRIDSEARCH module of CYANA. Calculations were initiated from 100 random conformers; the 10 structures with the lowest target functions were further analysed with the program MOLMOL [59].

### ThT fluorescence assay

The ThT solution (6.48 μL at 38.5 mM ThT in water) was added to 2.5 mL of the peptide stock solutions (the final concentrations of ThT and peptides were both 100 μM) at 25°C. For H3 mutA a similar experiment at 2 mM was also carried out and the sample was previously centrifuged. ThT fluorescence was measured using a Varian Cary Eclipse spectrofluorimeter (Varian, California, USA) and a cell consisting of a 10 × 10 mm path-length quartz cuvette, under magnetic stirring. Measurements were collected every day during 25–30 days, by using excitation and emission wavelengths of 440 and 450–600 nm, respectively.

### Dynamic light scattering (DLS)

H3 derived peptides (2 mM monomer concentration, and centrifuged), were kept under stirring in 50 mM phosphate, pH 7.2, at 25°C. The measurements were performed using a Zetasizer Nano S DLS device from Malvern Instruments (Malvern, Worcestershire, UK) with 633 nm laser, dual scattering angle mode, thermostated with a Peltier system and using a low-volume (45 μL), ultramicro cell. Size distributions by intensity and total light scattering intensity were determined in automatic mode at regular time-intervals over a period of 10 min for each measurement. Thirteen acquisitions were recorded, each 10 seconds in duration.

### Cell culture and transient transfection with synthetic peptides

Human SH-SY5Y neuroblastoma cells (A.T.C.C., Manassas, VA) were cultured in DMEM, F-12 Ham with 25 mM HEPES and NaHCO_3_ (1:1) and supplemented with 10% vol/vol FBS, 1.0 mM glutamine and antibiotics. Cell cultures were maintained in a 5.0% vol/vol CO_2_ humidified atmosphere at 37°C and grown until they reached 80% confluence for a maximum of 20 passages.

HeLa cells were grown in DMEM (Dulbecco's modified Eagle's medium) supplemented with 10% v/v of fetal bovine serum (FBS), 100 U/mL penicillin and 100 μg/mL streptomycin sulfate.

For transient peptide transfection, HeLa cells were seeded onto glass coverslip at 1 × 10^5^ cells/mL in 24-well plates overnight. The following day, 10 μM of TAT-H3 derived peptides were diluted in DMEM without red phenol and sonicated with a Bioruptor instrument (Diagenode, Liege, Belgium), 30 sec on/off at the highest power for 30 min to avoid aggregates and then incubated over cells for 24 h.

### MTT reduction assay

SH-SY5Y cells were seeded in 96-well plates. Aggregates of the H3 wt, H3 mutA and H3 mutE peptides (1 mM monomer concentration) were incubated in 50 mM sodium phosphate buffer, pH 7.2, 25°C under stirring, for 3 different times (0 h, 15 days and 30 days). The samples were then diluted into cell culture media at a 100 μM peptide concentration, and then added to the SH-SY5Y cells for 24 h at 37°C. Cell viability was then assessed by the 3-(4,5-dimethylthiazol-2-yl)-2,5-diphenyltetrazolium bromide (MTT) reduction assay as previously described [27]. Cell viability was expressed as the percentage of MTT reduction in treated cells compared to untreated cells (taken as 100%).

### Measurement of intracellular Ca^2+^

Aggregates of the H3 wt, H3 mutA and H3 mutE peptides (1 mM monomer concentration) were incubated in 50 mM sodium phosphate buffer, pH 7.2, 25°C under stirring, for 0 h, 15 days and 30 days. The samples were then diluted in cell culture media at a 100 μM peptide concentration, and then added to the SH-SY5Y cells seeded on glass coverslips for 60 min at 37°C. The cells were then loaded for 30 min at 37°C with 10 μM fluo3-AM (Life technologies, CA, USA), as previously described [27]. The resulting cell fluorescence was analysed by confocal Leica TCS SP5 scanning microscope (Mannheim, Germany) equipped with an argon laser source for fluorescence measurements at 488 nm and a Leica Plan Apo 63× oil immersion objective. A series of optical sections (1024 × 1024 pixels) 1.0 μm in thickness was taken through the cell depth for each examined sample. The confocal microscope was set at optimal acquisition conditions, e.g. pinhole diameters, detector gain and laser powers. Settings were maintained constant for each analysis. To quantify the signal intensity of the fluorescent probe between 10 and 22 cells were analysed using ImageJ software (NIH, Bethesda, MD) and the fluorescence intensities expressed as arbitrary units.

### Detection of subcellular localization

For fluorescence analyses, HeLa cells grown on glass coverslips were fixed with paraformaldehyde 4% v/v in PBS for 20 min at room temperature (RT). Fixed cells were washed with PBS and incubated with the TO-PRO-3 for nuclei counterstaining. After washing three times with Tween-20 0.1% v/v in TBS, the coverslips were mounted. Cells were examined under a Leica TCS SP laser-scanning confocal microscope (Leica Microsystems, Wetzlar, Germany) equipped with a 488-nm argon laser, a 543-nm He-Ne laser, and a 63× oil immersion objective (HCX PL APO CS 63×/1.32–0.60; Leica). Data were acquired at room temperature (23°C) using the integrated Leica Confocal Software package; multicolor images were captured through sequential scanning.

## SUPPLEMENTARY MATERIALS FIGURES AND TABLES



## Abbreviations

TIS: Triisopropylsilane
TFA: Trifluoroacetic acid
DMF: Dimethylformamide
HBTU: 1-H-Benzotriazolium 1-[bis(dimethylamino)methylene] Hexafluorophosphate(1-);3 oxide
DIEA: Di-isopropylethylamine
Fmoc: Fluorenylmethoxycarbonyl
HPLC: High Performance Liquid Chromatography
LC-MS: Liquid Chromatography Mass Spectrometry
NOESY: Nuclear Overhauser Enhancement Spectroscopy
TOCSY: Total Correlation Spectroscopy
CD: Circular Dichroism
CTD: C-Terminal Domain
NPM1: Nucleophosmin
MTT: 3-(4,5-dimethylthiazol-2-yl)-2,5-diphenyltetrazolium bromide
AML: acute myeloid leukemia
ThT: Thioflavin T
NES: nuclear export signal
NoLS: nucleolar localization signal
DLS: dynamic light scattering
NMR: nuclear magnetic resonance
CPP: cell penetrating peptide
