# Supplementary material for: Destabilisation, aggregation, toxicity and cytosolic mislocalisation of nucleophosmin regions associated with acute myeloid leukemia
Source: Oncotarget. 2016 Aug 1;7(37):59129–43. doi: 10.18632/oncotarget.10991 (PMC5312300; doi:10.18632/oncotarget.10991)
Supplement: Supplementary file 1 [file oncotarget-07-59129-s001.pdf]

Destabilisation, aggregation, toxicity and cytosolic mislocalisation of nucleophosmin regions associated with acute myeloid leukemia

Supplementary Materials

Supplementary Table S1: Chemical shifts (ppm) of the H3 wt peptide in 10 mM sodium phosphate buffer at pH 7.2

Residue	H_N	Hα	Hβ	Hγ	Others
**279 T**	8.26	4.34	4.28	1.22	
**280 D**	8.51	4.59	2.69–2.73		
**281 Q**	8.29	4.24	1.98–2.10	2.34	
**282 E**	8.33	4.18	1.97–2.03	2.27	
**283 A**	8.16	4.29	1.38		
**284 I**	7.96	4.065	1.86	1.46–1.16 γCH3 0.79	δCH3 0.87
**285 Q**	8.30	4.23	1.98–2.08	2.34	
**286 D**	8.27	4.58	2.62–2.67		
**287 L**	8.10	4.19	1.49–1.46		δCH3 0.76–0.85
**288 W**	8.10	4.51	3.29–3.26		Hε1 10.18 Hδ1 7.27
**289 Q**	8.07	3.97	1.86	2.034	
**290 W**	7.93	4.53	3.33–3.29		Hε1 10.14 Hδ1 7.27
**291 R**	7.90	3.99	1.74–1.64	1.45–1.38	Hδ3.03
**292 K**	8.03	4.13	1.74	1.38	Hε 2.95 Hδ1.64
**293 S**	8.11	4.37	3.89		
**294 L**	7.97	4.27	1.60		δCH3 0.80

Supplementary Table S2: Chemical shifts (ppm) of the H3 mutE peptide in 10 mM sodium phosphate buffer at pH 7.2

Residue	H_N	Hα	Hβ	Hγ	Others
**279 T**	8.27	4.35	4.28	1.23	Acetyl
**280 D**	8.52	4.61	2.73–2.70		
**281 Q**	8.30	4.28	2.14–1.99	2.38	
**282 E**	8.35	4.22	2.03–1.98	2.31	
**283 A**	8.19	4.32	1.40		
**284 I**	8.02	4.08	1.88	γCH3 0.88<br>1.49–1.19	δCH3 0.83
**285 Q**	8.34	4.24	1.97–2.00	2.35	
**286 D**	8.30	4.58	2.62–2.66		
**287 L**	8.09	4.18	1.50	1.50	0.81–0.86
**288 W**	8.08	4.57	3.34–3.38		Hε1 10.18<br>Hδ1 7.34
**289 Q**	8.03	4.15	1.94	2.18	
**290 S**	8.145	4.33	3.93		
**291 L**	8.139	4.314			
**292 A**	8.07	4.258	1.39		
**293 Q**	8.05	4.27	2.06	2.39	
**294 V**	8.02	4.00	2.14	0.96–0.99	
**295 S**	8.24	4.41	3.90		
**296 L**	8.07	4.30	1.69	1.69	
**297 R**	8.13	4.29	1.82–1.84	1.66–1.73	Hδ 3.20
**298 K**		4.28	1.81–1.86	1.45–1.50	Hε 3.01<br>Hδ 1.69

Supplementary Table S3: Chemical shifts (ppm) of the H3 mutA peptide in 10 mM sodium phosphate buffer at pH 7.2

Residue	H_N	Hα	Hβ	Hγ	Others
**279 T**	8.268	4.35	4.28	1.22	
**280 D**	8.53	4.62	2.70–2.73		
**281 Q**		4.30			
**282 E**		4.29	2.03–2.08	2.29	
**283 A**		4.37	1.41		
**284 I**	8.10	4.12	1.89	γCH3 0.93<br>1.49–1.21	δCH3 0.88
**285 Q**	8.43	4.29	2.04	2.33	
**286 D**	8.37	4.60	2.62–2.73		
**287 L**	8.23	4.31	1.66*	1.66	0.98
**288 C**	8.43	4.43	2.95		
**289 L**	8.17	4.33	1.65	1.65	0.98
**290 A**	8.24	4.37	1.41		
**291 V**	8.11	4.10	2.10	0.94	
**292 E**	8.52	4.29	1.97–2.04	2.28	
**293 E**	8.38	4.26	2.03	2.30	
**294 V**	8.17	4.00	2.13	0.97	
**295 S**	8.31	4.38	3.89		
**296 L**		4.32			
**297 R**	8.09	4.31	1.85	1.68	Hδ 3.21
**298 K**		4.30	1.86–1.81	1.70	Hδ 1.43–1.47<br>Hε 3.02

*Ambiguous chemical shifts due to spectral overlaps are colored red.

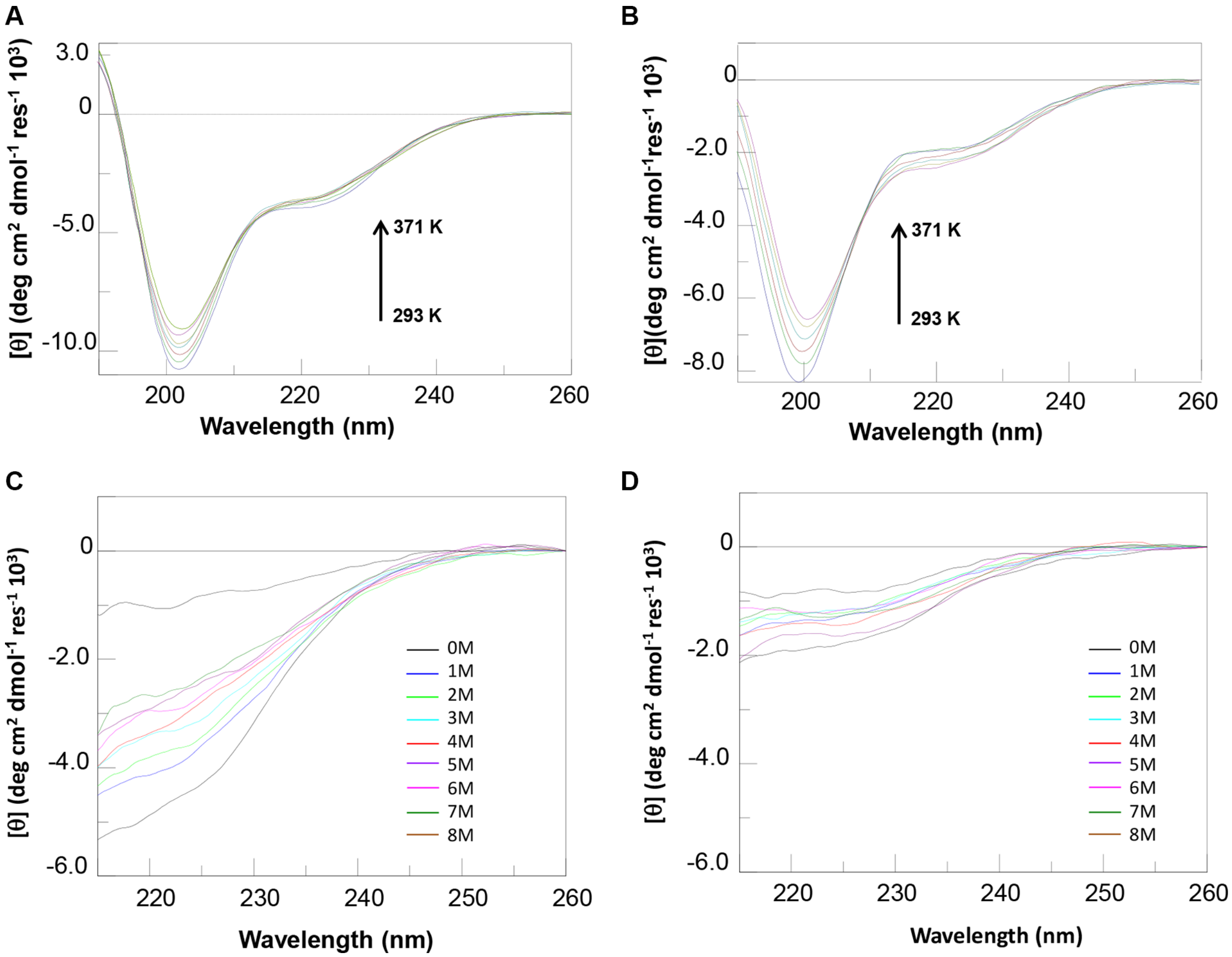

Supplementary Figure S1: CD spectra of H3 peptides. Overlay of CD spectra of the H3 mutE (**A**, **C**) and H3 mutA (**B**, **D**) peptides at increasing temperature (**A**, **B**) and urea concentration (**C**, **D**).

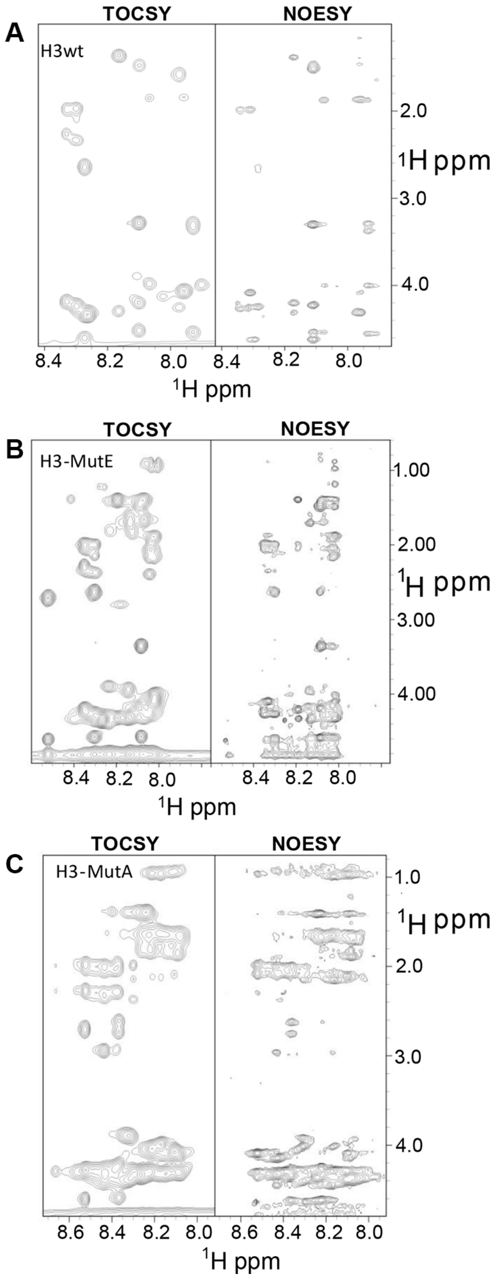

Supplementary Figure S2: NMR spectra of H3 peptides. Comparisons of 2D [^{1}H, ^{1}H] TOCSY (left panels) and NOESY 300 (right panels) spectra of (**A**) H3 wt, (**B**) H3 mutE, (**C**) H3 mutA peptides. The H_N /high field correlation regions of the spectra are reported in figure.

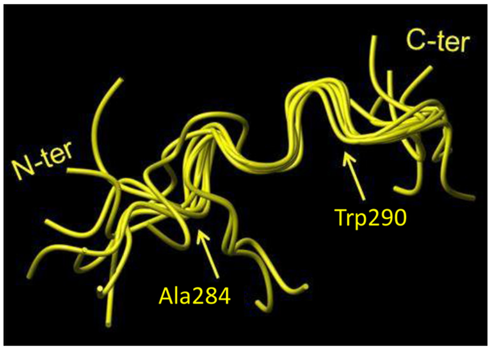

Supplementary Figure S3: NMR Structure of the H3 wt peptide. Superposition on the backbone atoms (residues Gln285-Arg291: RMSD = 0.48 Å) of 10 conformers. Structure calculations included 73 distance constraints (27 intra-residue, 31 short-range, 15 medium-range) and 89 angle constraints.

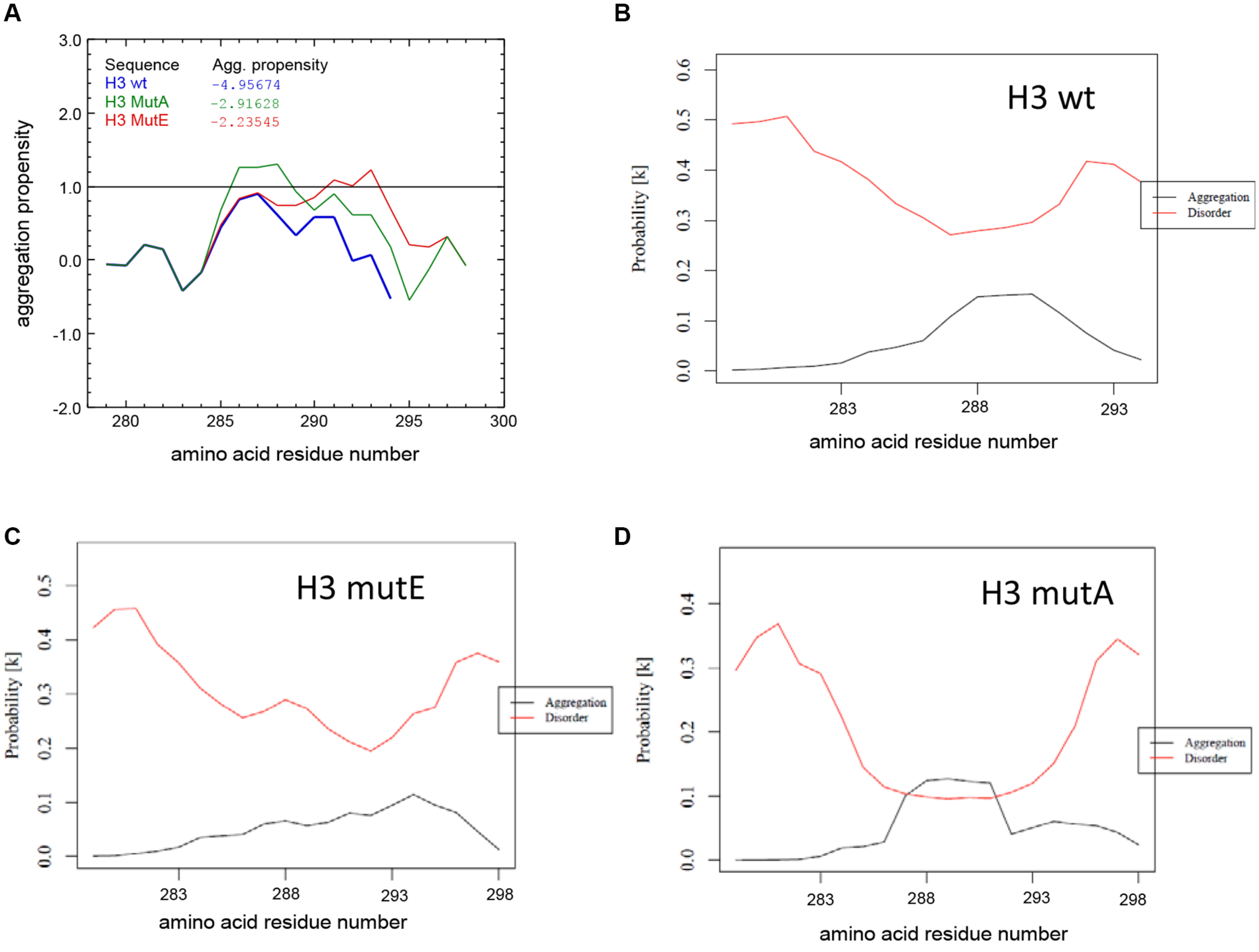

Supplementary Figure S4: Aggregation propensity profiles of H3-derived peptides. (**A**) Aggregation propensity profiles using Zyggregator. The horizontal line at a value of 1 indicates a threshold between aggregation-promoting regions (above the line) and soluble regions (below the line). (**B–D**) Probability of amyloid-like aggregation versus disorder determined using PASTA for the (B) H3 wt, (C) H3 mutE, (D) H3 mutA peptides.

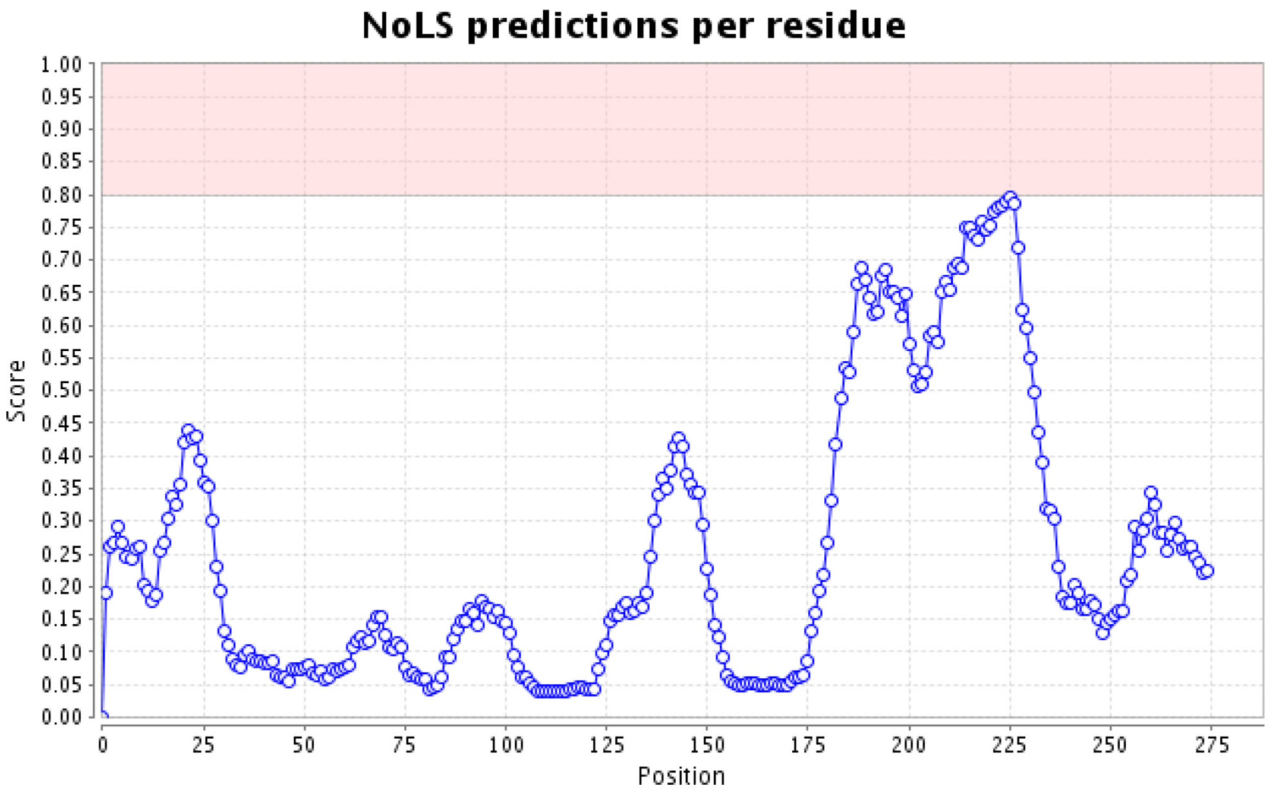

Supplementary Figure S5: Predicted NoLSs in the sequence of NPM1. Prediction of NoLSs in ful-length B23.1 using the NOD program (http://www.compbio.dundee.ac.uk/nod).
